# Musculoskeletal disorders in Norway: prevalence of chronicity and use of primary and specialist health care services

**DOI:** 10.1186/s12891-015-0536-z

**Published:** 2015-04-02

**Authors:** Jonas Minet Kinge, Ann Kristin Knudsen, Vegard Skirbekk, Stein Emil Vollset

**Affiliations:** Norwegian Institute of Public Health, Postboks 4404 Nydalen, 0403 Oslo, Norway; Department of Health Management and Health Economics, University of Oslo, Boks 1072, 0316 Oslo, Norway; Department of Global Public Health and Primary Care, University of Bergen, Postboks 7800, 5020 Bergen, Norway; Columbia Aging Center, Columbia University, 722 W. 168th Street, New York, NY 10032 USA

**Keywords:** Musculoskeletal disorders, Prevalence, Health services, Primary health care, Specialist health services, Norway

## Abstract

**Background:**

Uncertainty exists with regards to the extent of prevalence and health care use for musculoskeletal disorders in Norway. The aim of this study was to estimate the prevalence of chronic musculoskeletal disorders and to estimate the prevalence of persons receiving primary and specialist health services for these disorders.

**Methods:**

We used three data-sources. First, four discrete years of the nationally representative cross-sectional Survey of Health and Living Conditions (SHLC) conducted in 2002, 2005, 2008 and 2012 by Statistics Norway. Second, we used the Norwegian Patient Registry (NPR) to estimate the proportion of the population who used specialist health services in 2012. Third, we used the national register dataset for reimbursement of primary care physicians, chiropractors and physiotherapists (KUHR) to estimate the proportion of the population attending primary care physicians, chiropractors or physiotherapists in 2012. Age- and sex-specific prevalence/utilization estimates for musculoskeletal disorders were calculated.

**Results:**

In 2012, 18% of men and 27% of women reported musculoskeletal disorders lasting for six months or more in the SHLC. Primary health care services reimbursed for musculoskeletal disorders were used by 37% of women and 30% of men. Of these 32% (women) and 26% (men) were physician contacts and between 5 and 9% physiotherapist or chiropractor or combined contact types. Corresponding numbers for specialist services were 5% in men and 7% in women, where the majority was out-patient consultations. Low back and neck pain were the most common diagnoses both in the general population and as reason for health care utilization. We found that musculoskeletal disorders increased with age, however our results showed no variation in prevalence of chronic disorders between 2002 and 2012.

**Conclusion:**

Chronic musculoskeletal disorders were common in the general population, with higher prevalence among women compared to men, and increasing prevalence with age. Musculoskeletal disorders had considerable impact on the use of primary and specialist health services in Norway. The use of register data on health service utilization may be a useful source for monitoring population trends, and for estimating the burden in terms of health and health service use.

**Electronic supplementary material:**

The online version of this article (doi:10.1186/s12891-015-0536-z) contains supplementary material, which is available to authorized users.

## Background

Musculoskeletal disorders constitute a major health challenge for individuals, health systems, and social care systems across the globe [1,2]. According to Eurostat, musculoskeletal disorders are the most costly work-related European health problem, affecting about 45 million workers [3]. Studies suggest that this health challenge has been increasing [4] and it is predicted to continue to increase in the future [1]. There are reasons to suggest that the extent of the problem is particularly high in Norway. Norwegian national statistics show that musculoskeletal disorders are the most common cause for sick leave, disability retirement and for attending primary care [5]. In addition, low back pain and neck pain are leading causes of Disability Adjusted Life Years (DALYs) in Norway, as well as in other western countries, as estimated by the Global Burden of Disease (GBD) study [6,7]. In fact, Norway was the country in the world with the greatest burden of disease from musculoskeletal disorders estimated in GBD 2010 [6].

The GBD estimates on disease burden in Norway due to musculoskeletal disorders are, however, plagued with the uncertainty due to a large spread in prevalence estimates. This large variability is primarily related to heterogeneity in the measurement and definition of musculoskeletal disorders. Comparability between the studies is difficult as both the duration of the disorder, as well as the reference period (last week, last month or last year), is essential for estimating prevalence. Population studies have estimated prevalence of overall musculoskeletal disorders ranging from 23-80% among adults in Norway [4,8-10]. For example, while 75-80% has any sort of pain in the musculoskeletal system in a month in Norway [9], 45–48% reported musculoskeletal disorders lasting more than three months during the past year [4,10]. Furthermore, there are differences in the definitions applied and the focus of the studies varies widely. Some studies have assessed self-reported general pain in musculoskeletal system, while others have studied one or more specific diagnoses [10]. Also, many studies, especially in Norway, have been conducted in highly selected subpopulations such as hospitalized patients [11], restricted age groups [12-14], females [13,14] or have been geographically constrained to a specific area within a country [4,10,13-17]. Up-to-date information about the levels and trends is essential to quantify the extent of the problem, and to motivate decision makers to fund research and treatment for musculoskeletal disorders.

The aim of the current study was to describe the prevalence (existing cases) of musculoskeletal disorders in the general Norwegian population and the use of primary and specialist health services for these disorders. This is the first study to use both nationwide health registers and health survey data to estimate the proportion and resulting health service use from musculoskeletal disorders in Norway. By combining different data sources we contribute to a more detailed insight into how specific types of musculoskeletal disorders have different prevalence levels in Norway. These results can be used as input to overall prevalence estimates of musculoskeletal disorder in later studies.

## Methods

### Data

Three data-sources were employed in the present study. We used data from four waves of the Survey of Health and Living Conditions (SHLC), which is a cross-sectional health survey, with data collected in 2002, 2005, 2008 and 2012 by Statistics Norway [18]. The survey is a nationally representative health survey of individuals in Norway over the age of 15. Statistics Norway draws a random sample of 10 000 individuals each year and then exclude individuals who have died or have moved abroad. In addition, individuals living in the following institutions are excluded from the data-collection: retirement homes, combined hospitals and retirement homes, orphanages, youth homes, psychiatric hospitals and nursing homes, institutions for those with developmental disabilities, and institutions for individuals with alcohol- and drug use disorders. The exclusion of these individuals sum up to around 200 individuals each year, thus individuals with severe mental and somatic health problems who are not able to live independently, and the oldest age-groups are under-represented in this survey.

The part of the health survey used in the present study was based on telephone interviews, and an extensive effort was made in advance of the interviews to obtain a telephone number for each individual (mobile or home telephone). However, home interviews were also conducted in certain settings. Of those who were contacted for the telephone interview 71%, 70%, 67% and 58% participated in 2002, 2005, 2008 and 2012, respectively. During the telephone interview individuals were asked if they have any longstanding (chronic) disabilities or disorders lasting over six months. The interviewer repeated the question until each disability was noted down; care was required so that each disability was reported only one time. They then gave a description for each longstanding illness, which were later given an ICD-10 code by Statistics Norway. In addition, the respondents were sent a card in advance of the phone interview with 57 diagnoses to help in answering the question about longstanding illnesses [18]. We created nine binary variables, each of which takes the value of one if the respondent was reporting a chronic disability within a specific diagnostic classification (Table 1) and zero otherwise.

**Table 1:** **Disease categories by ICD-10 codes and ICPC-2 codes**

	**ICD-10** ^**1**^	**ICPC-2**
Low back and neck pain	M46.9, M47, M48.0-M48.2, M48.8-M48.9, M50-M54	L01, L02, L03, L84, L86, L85, L83
Shoulder syndrome	M75	L08, L92
Osteoarthritis	M15-M19	L89, L90, L91
Osteoporosis	M80-M81	L95
Rheumatoid arthritis*	M05-M06	L88
Fibromyalgia	M79	L18
Bursitis/tendinitis/synovitis	M65–M67, M70–M72, M760–M770, M772–M779	L87
Gout	M10	T92
Total musculoskeletal	All M codes	All L codes

*In ICPC-2 this category is defined as “Rheumatoid/seropositive arthritis”.

^1^Based on GBD cause list [19].

The second data-source was a complete population based nationally administrative register dataset for reimbursement of primary care physicians, chiropractors and physiotherapists (KUHR) for 2012. This is a register consisting of all reimbursement records sent by primary care physicians, chiropractors and physiotherapists in Norway. In Norway physicians, chiropractors and physiotherapists are required to submit at least one ICPC-2 code per patient treated to order to be reimbursed. This means that the unit of registration is the number of reimbursement claims by ICPC-2 codes. We note that if a patient was treated for multiple diseases in a single attendance, the physicians are only required to submit one ICPC-2 code to be reimbursed. However, they can choose to submit multiple codes. The data, which was stratified by age and gender, measure musculoskeletal disorders in primary care. Whether a person is registered with a diagnosis is conditional on 1) that the person has contacted primary care and 2) that a physician, chiropractor or physiotherapist defines it as musculoskeletal disorders.

Finally, we used data from the Norwegian Patient Registry (NPR) in 2012, which is a complete population based nationally administrative health register which covers all inpatient, day patient and outpatient specialist health services in Norway. As the NPR is the basis for funding of hospital and specialist services, it is compulsory to report diagnosed procedures for each contact. The NPR unit of registration is admissions to hospital and contact with the specialist health services and a new ICD-10 code is provided for each admission. The NPR dataset, which was stratified by age and gender, is a measure of musculoskeletal disorders in specialist health care (inpatient, day patient, outpatient consultation). Whether a person is registered with a diagnosis conditional on 1) that the person has been referred to specialist health care services by a primary care health care worker, and 2) that a physician reported it as a musculoskeletal disorder.

The definitions of each disorder category employed in the present study can be found in Table 1. The categories were defined based on the definitions and categorisations used in GBD 2010 [19], except from the category “all musculoskeletal disorders” which includes all ICD-10 codes in the M chapter and all ICPC-2 codes in the L-chapter. Although the M chapter and the L chapter are overlapping, they are not identical. For example, the L chapter includes musculoskeletal malignant neoplasms, fractures and injuries, which are not included in the M chapter.

In the SHLC most ICD-10 codes were given with only three characters. The definitions of “low back and neck pain” and “bursitis/tendinitis/synovitis” contain ICD-10 codes with four characters. Hence, in this case we might underestimate the prevalence. We have conducted analyses using three characters for these groups and the differences were not important for the main results.

In terms of duration of the disorders, we did only have an indication of this in the SHLC data, which has records for chronic musculoskeletal disorders lasting 6 months or more. Information about duration was not included in the KUHR and NPR databases.

The participants in SHLC gave their informed consent at the time of the telephone interview, and the usage of SHLC was approved by the Norwegian Social Science Data Services. The KUHR and NPR datasets were aggregated and anonymous. The usage of the data is in accordance with the regulations for KUHR and NPR and do not need approval from the Regional Ethics Committee.

### Statistical analysis

In the current analysis we estimated weighted mean prevalence of musculoskeletal disorders in the general population by age, gender and year based on the Survey of Health and Living Conditions. To account for missing responses in SHLC we used the sample weights provided in the survey. These were calculated based on gender, age, education and family size [18]. No sample weights were applied to the estimates from register datasets as they are complete representations for the Norwegian population.

We calculated binomial standard errors. The sampling strategy used in SHLC in 2002, 2005 and 2008 was that they randomly picked a number of sampling units across Norway. If we assume independence within these sampling units the standard errors will be too small. We therefore adjust for clustered sampling by a design factor of 2.05. In 2012 the observations were drawn randomly from the whole country, hence adjustment for clustering was not needed. The inclusion of four discrete years of the health survey allowed us to calculate age adjusted prevalence estimates and confidence intervals for comparison across time.

We calculated the fraction of the population who had a primary care visit or who had contact with the specialist services due to musculoskeletal disorder. This was done by dividing the absolute number of individuals who used each service in 2012 on the total number of individuals in Norway, by age-gender group. Data on number of individuals in Norway by age and gender was retrieved from the official census from Statistics Norway, and includes all persons registered as resident in Norway on the 1st of January each year, who resides in Norway for at least six months and who have a valid residence permit [20]. Hence, we present the share of the population registered in KUHR and NPR with one of the disorder categories as defined in Table 1. We also estimated binomial standard errors.

## Results

The total number of respondents in the SHLC across the four rounds between 2002 and 2012 was 25 718. Of these 5 828 (23%) reported chronic musculoskeletal disorders. The total Norwegian population in 2012 was 4.9 mill of which 292 610 (6%) had at least one specialist (day-, in-, or outpatient) contact for musculoskeletal disorders, 613 708 (12%) had at least one chiropractor or physiotherapist visit, and 1.4 mill (29%) had at least one primary care physician visit for musculoskeletal disorders.

Table 2 shows that the prevalence of the Norwegian population reporting chronic musculoskeletal disorders was higher in women than in men in 2012. This was also reflected in health service use. Eighteen percent of the men and 27% of the women were registered with chronic disorders. Among men 5% of the population had contact with the specialist services and 30% had used primary care services for musculoskeletal disorders. Similar numbers among women were 7% and 37%. The most common chronic disorder was osteoarthritis in women and low back and neck pain in men. These were also the most common reasons for specialist contacts in men and women, respectively. However, low back and neck pain was, by far, the most common reason for primary care use in both genders. We also see that the share of the Norwegian population who were registered with primary care visits for musculoskeletal disorders were higher than the prevalence estimate for chronic musculoskeletal disorders in the SHLC.

**Table 2:** **Prevalence estimates (%) based on individuals who reported a chronic condition(s), had one or more contacts with specialist health services (hospital or specialist out-patient) or primary care (physician or physiotherapist or chiropractor) for musculoskeletal disorders in survey of health and living conditions, NPR and KUHR in 2012**

	**Chronic disorders** ^**1**^			**Specialist health services** ^**2**^			**Primary care** ^**3**^		
	**Prevalence (%)**	**S.E.**	**Freq.**	**Prevalence (%)**	**S.E.**	**Freq.**	**Prevalence (%)**	**S.E.**	**Freq.**
**Men**	***(N = 2807)***			***(N = 2498871)***			***(N = 2498871)***		
Low back and neck pain	7.55	0.49	203	1.08	0.01	26874	17.62	0.02	440345
Shoulder syndrome	0.00	0.00	0	0.57	0.01	14335	3.99	0.01	99753
Osteoarthritis	5.46	0.43	156	0.97	0.01	24180	1.98	0.01	49599
Osteoporosis	0.31	0.11	10	0.07	<0.01	1658	0.11	<0.01	2658
Rheumatoid arthritis*	1.17	0.19	28	0.23	<0.01	5779	0.65	0.01	16279
Fibromyalgia	2.67	0.31	77	0.51	0.01	12823	1.10	0.01	27364
Bursitis/tendinitis/synovitis	0.68	0.16	21	0.53	0.01	13278	2.26	0.01	56392
Gout	0.54	0.14	15	0.09	<0.01	2213	0.70	0.01	17497
Total musculoskeletal	18.43	0.73	518	5.01	0.01	125059	30.05	0.03	750899
**Women**	***(N = 2853)***			***(N = 2486999)***			***(N = 2486999)***		
Low back and neck pain	7.92	0.50	220	1.20	0.01	29843	21.79	0.03	541909
Shoulder syndrome	0.00	0.00	0	0.53	0.01	13125	4.75	0.01	118252
Osteoarthritis	11.20	0.59	323	1.58	0.01	39162	4.17	0.01	103794
Osteoporosis	3.43	0.34	97	0.36	<0.01	8901	0.96	0.01	23816
Rheumatoid arthritis*	2.19	0.27	60	0.58	0.01	14432	1.24	0.01	30784
Fibromyalgia	6.34	0.46	180	0.83	0.01	20507	3.33	0.01	82729
Bursitis/tendinitis/synovitis	0.77	0.17	23	0.59	0.01	14692	3.05	0.01	75783
Gout	0.39	0.11	9	0.02	<0.01	584	0.22	<0.01	5486
Total musculoskeletal	27.43	0.83	778	6.74	0.02	167551	37.00	0.03	920261

^1^ICD-10 codes: M-chapter (Health survey SHLC 2012).

^2^ICD-10 codes: M-chapter (NPR 2012).

^3^ICPC-2 codes: L-chapter (KUHR 2012).

*In ICPC-2 this category is defined as “Rheumatoid/seropositive arthritis”.

Note: The means in in the survey of health and living conditions are weighted. The standard errors (S.E.) are based on a binomial distribution.

Comparing women and men, we see that low back pain, osteoarthritis, osteoporosis, rheumatoid arthritis, fibromyalgia, and bursitis/tendinitis/synovitis were more common in women across each data source, while gout was more common in men across each data source. Shoulder syndrome was more common in women than men in primary care; however, men had more specialist contacts for shoulder syndrome.

Table 3 shows that the prevalence of the population who used outpatient services for musculoskeletal disorders were higher than the prevalence of the population who used day patient and inpatient services. Hence, the majority of the specialist health services used for musculoskeletal disorders was outpatient services for both men and women.

**Table 3:** **Prevalence estimates (%) of specialist services use for musculoskeletal disorders**

	**Day patient**			**Inpatient**			**Outpatient**			**Total N**
***Gender***	***Prevalence (%)***	***S.E.***	***Freq.***	***Prevalence (%)***	***S.E.***	***Freq.***	***Prevalence (%)***	***S.E.***	***Freq.***	
Men	0.67	0.01	16720	0.54	0.01	13416	3.80	0.01	94923	2498871
Women	0.70	0.01	17425	0.64	0.01	16047	5.37	0.01	134079	2486999

Table 4 shows that 26% of men and 32% of women visited a primary care physician for musculoskeletal disorders in 2012. Six percent of men and women had a seen a chiropractor while 5% of men and 9% of women had seen a physiotherapist for musculoskeletal disorders.

**Table 4:** **Prevalence estimates (%) of primary care use for musculoskeletal disorders**

	**Physician**			**Chiropractor**			**Physiotherapist**			**Total N**
***Gender***	***Prevalence (%)***	***S.E.***	***Freq.***	***Prevalence (%)***	***S.E.***	***Freq.***	***Prevalence (%)***	***S.E.***	***Freq.***	
Men	25.596	0.028	639613	5.86	0.02	146321	5.09	0.01	127058	2498871
Women	32.260	0.030	802309	6.36	0.02	158221	8.81	0.02	219067	2486999

**Figure 1:**
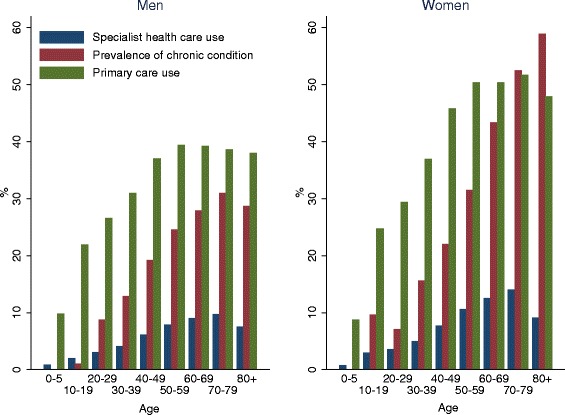
**Prevalence estimates (%) based on individuals who reported a chronic condition(s), had one or more contacts with specialist health services (hospital or specialist out-patient) or primary care (physician or physiotherapist or chiropractor) for musculoskeletal disorders in survey of health and living conditions, NPR and KUHR in 2012, by age groups.** No data exists for those under the age of 15 in the Survey of Health and Living Conditions.

Figure 1 illustrates the mean prevalence and the mean use of primary care and specialist services in 2012 for total musculoskeletal disorders by age groups in Norway. This figure demonstrates the age and gender pattern in musculoskeletal disorders. Both the estimated prevalence and the use of relevant services increased sharply by age. Women have a higher prevalence across all age groups, except for those under the age of 10. In addition, the difference between men and women increased by age.

Figure 1 also illustrates that the age and gender pattern followed each other across the three data sources, except from in older age groups in women. The prevalence of chronic musculoskeletal disorders increased in women aged 80+, however the use of both primary and specialist care services decreased, probably because most health service use in this age-group takes place within nursing homes for the elderly or similar institutions.

**Figure 2:**
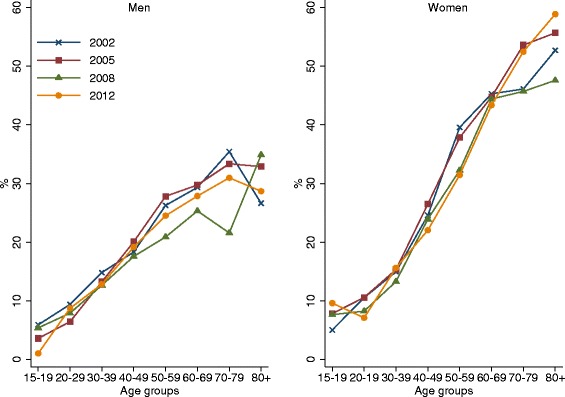
**Prevalence estimates (%) of chronic musculoskeletal disorders in men and women in survey of health and living conditions by age and year.**

Table 5 shows the mean prevalence of chronic musculoskeletal disorders measured in the four waves of SHLC in men and women by year. There were minor variations across time in chronic musculoskeletal disorders in Norway in both men and women. We observe a slightly decreased prevalence; however the difference between 2002 and 2012 was not significant, and could be the result of increased selective nonparticipation among individuals with chronic musculoskeletal disorders across the waves. Figure 2 shows the prevalence of chronic musculoskeletal disorders across age, gender and year in the SHLC. The figure shows that the age pattern identified in Figure 1 has been consistent between 2002 and 2012.

**Table 5:** **Age adjusted prevalence estimates (%) of chronic musculoskeletal disorders in men and women in survey of health and living conditions by year**

	**Men**				**Women**			
***Year***	***Prevalence (%)***	***S.E.***	***Freq.***	***N***	***Prevalence (%)***	***S.E.***	***Freq.***	***N***
2002	19.57	0.97	664	3410	28.21	1.09	918	3417
2005	19.54	0.97	651	3401	29.05	1.11	936	3365
2008	16.92	0.95	537	3172	26.13	1.08	826	3293
2012	18.43	0.73	518	2807	27.43	0.83	778	2853

Note: The means are weighted. The standard errors (S.E.) are based on a binomial distribution and are corrected for clustered sampling in 2002, 2005 and 2008.

Additional file 1: Tables A.1 and A.2 show in more detail the prevalence and use of health services by year, age and gender from total musculoskeletal disorders. Additional file 1: Tables A.3 and A.4 show the prevalence and use of health services by year, age and gender from low back and neck pain (Additional file 1).

## Discussion

This study used three separate data sources to estimate the prevalence of chronic musculoskeletal disorders and the prevalence of individuals attending primary and specialist health services for these disorders in Norway. We found that chronic musculoskeletal disorders were common in the general population. Among both men and women, the population prevalence and health service utilization increased with age. The burden was higher in women and the gender disparities for all of our measures increased with age. There was no variation in the prevalence between 2002 and 2012 and the age pattern was consistent in each year.

Direct comparison between the prevalence estimates found in our study and in those of earlier studies is difficult due to the variation in study methodology, e.g., time frame, age range, definitions used and data collection methods. As far as we know, no other study has employed data collected with comparable methods to ours in terms of time frame and source of registration. In general, our findings are consistent with studies that show that the prevalence of common musculoskeletal disorders increase with age [1,4] and that women are more affected than men [4,17]. However, Hagen et al. [4] found an increasing prevalence of musculoskeletal disorders in Nord-Trønderlag county in Norway between 1995–97 and 2006–08. We find a non-significant decreased prevalence between 2002 and 2012 using the nationally representative Survey of Health and Living Conditions. There may be a number of reasons for our conflicting findings. Firstly, while our dataset was nationally representative of the Norwegian population, Hagen et al. [4] used data from only one of 19 Norwegian counties. We also used different measures of musculoskeletal disorders. Hagen et al. [4] identified chronic musculoskeletal disorders by a screening question directly on pain/stiffness in muscle or joints lasting more than three months, while in our dataset (SHLC) participants were asked to describe any longstanding (chronic) disabilities or disorders lasting over six months. In addition, the survey response rates in Hagen et al. [4] were 70% in 1995–97 and 42% in 2006–08, while our survey response rates were 71%, 70%, 67% and 58% in 2002, 2005, 2008 and 2012, respectively. Furthermore, we used more recent data. However, more research using multiple data sources is needed to investigate this further.

Our findings showed that osteoarthritis was the most common chronic musculoskeletal disorder reported by women. This might be related to the duration of chronicity applied in the Survey of Health and Living Conditions. Osteoarthritis is a chronic condition, while, for example, low back and neck pain often are more temporary. Hence, if a shorter time frame were used in this study, low back and neck might have been more common than osteoarthritis in Norwegian women. This was supported by the findings showing that low back and neck pain was by far the most common reason for primary care utilization. The six month time frame might also be the reason for why we find that a higher share of the Norwegian population have utilized primary care physicians due to musculoskeletal disorders, than the prevalence of musculoskeletal disorders reported in the general population.

The variation across age and gender in musculoskeletal disorders, based on the cross-sectional health survey, and data on use of primary care and specialist services, from national registers, followed each other closely. This suggests that register data on primary care consultations in combination with specialist services can be a useful source to examine variations in the true population prevalence in Norway. However, further investigations with linkage of register data at the individual level are needed.

Our findings also suggest that musculoskeletal disorders are highly age and gender dependent. The population in Norway is aging [21] and the population aged 70 or more will grow rapidly in the coming years. This age-group is projected to double in size in the next 30 years as a consequence of the post-war baby boomers growing older [22]. The strong association between prevalence and age means that musculoskeletal disorders is likely to be an increasing public health challenge in Norway in the future. However, slow progress is being made in understanding how to manage the impact of these disorders on population health and the public health care system [23]. This underscores the need for cost-effectiveness analyses in this field, in which reliable data is needed on prevalence and use of health services.

A strength of this study was the use of two nationwide registries combined with nationally representative survey data. In addition, it is an advantage that the same question- focusing on chronic disorders- has been asked in four cycles over an 11 year time period. This ensures consistency in the measurement. It is also an advantage that the register datasets used in this study are without missing responses as musculoskeletal disorders are more common among nonparticipants in health surveys [24]. However, the register data do not measure population disease prevalence. The estimates will not pick up individuals who have musculoskeletal disorders without consulting primary care or specialist health services.

We note that there is a likely overlap between the sources, individuals who report chronic musculoskeletal disorders may also visited their primary care physician and subsequently have a procedure in the specialist health services. In this way the same individuals may be picked up in each of the data sources we used. This means that we cannot add up the sources to get an overall prevalence. Future studies would benefit from employing individual linkages from health surveys to health registries, and by that being able to follow the health service trajectory of an individual who report musculoskeletal disorders in a health survey.

This study has a number of limitations that must be considered when interpreting the results. There may be measurement error and reporting bias. The reporting of chronic illnesses in the Survey of Health and Living Conditions is self-reported and may therefore suffer from systematic bias, which might be related to age and gender. The register data might also suffer from reporting bias for a number of reasons. A diagnosis is the key to various social benefits in Norway [25] and many of the musculoskeletal disorders are essentially subjective. Hence, despite having the same symptoms, patients may be labelled differently by different physicians, and in different countries depending on the social security system [25]. This may be an issue for further research, where for example cross-country data on different symptoms and resulting diagnoses could be applied. In addition, the use of primary care services in KUHR may be biased due to systematic under-reporting. The primary care physician, chiropractor and physiotherapist are only obliged to provide one diagnosis for the KUHR in order to be reimbursed. If a patient is treated for a number of illnesses the physician can choose to only report one. These concerns might be especially important in the field of musculoskeletal disorders, in which there is a large overlap between health symptoms, other pain conditions (i.e. headache, gastrointestinal complaints etc.) and with mental health problems. Further research could explore this issue by linking the different data sources to explore whether multiple conditions are underreported, or by following individuals over an extended time period and examine the number of different diagnoses given to the same individual. Finally, although the nature of the disorders in terms of diagnostic categories was comparable between the data-sources, we only had information about the chronicity of the disorders from the SHLC (>6 months). Thus, the duration and hence the severity of the disorders may differ somewhat between the data-sources, although we believe that the disorder may be more than ignorable to be treated in the health care service.

## Conclusion

The present analysis revealed that musculoskeletal disorders constituted an important burden on the population and the Norwegian health care system between 2002 and 2012. There was a clear age gradient where the prevalence and use of health care services was higher among older men and women. Musculoskeletal disorders were more common in women than in men.

## Additional file

Additional file 1: Table A.1-A.4. The file contains the prevalence and health service use for musculoskeletal disorders by age for men and women. The file also contains the prevalence and health service use for lower back and neck pain by age for men and women.
