# Supplementary material for: Musculoskeletal disorders in Norway: prevalence of chronicity and use of primary and specialist health care services
Source: BMC Musculoskelet Disord. 2015 Apr 2;16:75. doi: 10.1186/s12891-015-0536-z (PMC4392859; doi:10.1186/s12891-015-0536-z)
Supplement: Additional file 1: Table A.1-A.4. — The file contains the prevalence and health service use for musculoskeletal disorders by age for men and women. The file also contains the prevalence and health service use for lower back and neck pain by age for men and women. [file 12891_2015_536_MOESM1_ESM.docx]

*Additional file 1 A.1: Prevalence and health service use for musculoskeletal disorders by age in men*

|  | <10 |  |  |  |  | 10-19 |  |  |  |  | 20-29 |  |  |  |  | 30-39 |  |  |  |  | 40-49 |  |  |  |
| --- | --- | --- | --- | --- | --- | --- | --- | --- | --- | --- | --- | --- | --- | --- | --- | --- | --- | --- | --- | --- | --- | --- | --- | --- |
|  | *Mean (%)* | *S.E.* | *Freq.* | *N* |  | *Mean (%)* | *S.E.* | *Freq.* | *N* |  | *Mean (%)* | *S.E.* | *Freq.* | *N* |  | *Mean (%)* | *S.E.* | *Freq.* | *N* |  | *Mean (%)* | *S.E.* | *Freq.* | *N* |
| *Survey of health and living conditions* | | | | |  |  |  |  |  |  |  |  |  |  |  |  |  |  |  |  |  |  |  |  |
| 2002 | - | - | - | - |  | 5.90 | 2.24 | 13 | 224 |  | 9.39 | 1.82 | 50 | 529 |  | 14.82 | 1.96 | 97 | 666 |  | 18.32 | 2.14 | 123 | 672 |
| 2005 | - | - | - | - |  | 3.59 | 1.67 | 9 | 253 |  | 6.44 | 1.54 | 33 | 518 |  | 13.25 | 1.90 | 83 | 639 |  | 20.07 | 2.32 | 124 | 613 |
| 2008 | - | - | - | - |  | 5.39 | 2.18 | 10 | 203 |  | 7.89 | 1.79 | 35 | 456 |  | 12.66 | 2.03 | 64 | 528 |  | 17.57 | 2.16 | 110 | 631 |
| 2012 | - | - | - | - |  | 1.03 | 0.78 | 2 | 181 |  | 8.72 | 1.30 | 32 | 417 |  | 12.84 | 1.58 | 52 | 429 |  | 19.16 | 1.75 | 92 | 496 |
| GP visit 2012 | 6.92 | 0.05 | 21687 | 313388 |  | 19.54 | 0.07 | 63994 | 327534 |  | 22.66 | 0.07 | 75383 | 332710 |  | 25.43 | 0.07 | 88336 | 347331 |  | 30.92 | 0.08 | 115349 | 373083 |
| Chiro/Phys 2012* | 3.37 | 0.03 | 10575 | 313388 |  | 4.93 | 0.04 | 16161 | 327534 |  | 7.93 | 0.05 | 26377 | 332710 |  | 11.52 | 0.05 | 40023 | 347331 |  | 14.10 | 0.06 | 52616 | 373083 |
| GP/Chiro/Phys 2012** | 9.75 | 0.05 | 30553 | 313388 |  | 21.95 | 0.07 | 71883 | 327534 |  | 26.53 | 0.08 | 88266 | 332710 |  | 30.99 | 0.08 | 107654 | 347331 |  | 37.01 | 0.08 | 138081 | 373083 |
| NPR 2012 | 0.82 | 0.02 | 2567 | 313388 |  | 2.03 | 0.03 | 6651 | 327534 |  | 3.00 | 0.03 | 9965 | 332710 |  | 4.09 | 0.03 | 14204 | 347331 |  | 6.15 | 0.04 | 22929 | 373083 |
|  | 50-59 |  |  |  |  | 60-69 |  |  |  |  | 70-79 |  |  |  |  | 80+ |  |  |  |  |  |  |  |  |
| Survey of health and living conditions | | | | |  |  |  |  |  |  |  |  |  |  |  |  |  |  |  |  |  |  |  |  |
| 2002 | 26.27 | 2.58 | 155 | 593 |  | 29.34 | 3.36 | 112 | 379 |  | 35.43 | 4.37 | 86 | 245 |  | 26.63 | 6.33 | 28 | 102 |  |  |  |  |  |
| 2005 | 27.82 | 2.60 | 163 | 599 |  | 29.75 | 3.16 | 120 | 419 |  | 33.38 | 4.30 | 82 | 246 |  | 32.88 | 6.28 | 37 | 114 |  |  |  |  |  |
| 2008 | 20.88 | 2.51 | 111 | 536 |  | 25.32 | 2.84 | 113 | 466 |  | 21.58 | 3.97 | 49 | 223 |  | 34.89 | 6.01 | 45 | 129 |  |  |  |  |  |
| 2012 | 24.52 | 1.88 | 123 | 514 |  | 27.87 | 2.07 | 124 | 459 |  | 30.97 | 3.02 | 71 | 233 |  | 28.69 | 5.10 | 22 | 78 |  |  |  |  |  |
| GP visit 2012 | 33.86 | 0.08 | 108282 | 319785 |  | 34.23 | 0.09 | 91821 | 268253 |  | 34.08 | 0.13 | 46730 | 137117 |  | 35.18 | 0.17 | 28031 | 79670 |  |  |  |  |  |
| Chiro/Phys 2012* | 14.67 | 0.06 | 46918 | 319785 |  | 14.64 | 0.07 | 39267 | 268253 |  | 14.20 | 0.09 | 19466 | 137117 |  | 10.19 | 0.11 | 8118 | 79670 |  |  |  |  |  |
| GP/Chiro/Phys 2012** | 39.41 | 0.09 | 126035 | 319785 |  | 39.24 | 0.09 | 105254 | 268253 |  | 38.58 | 0.13 | 52894 | 137117 |  | 38.01 | 0.17 | 30279 | 79670 |  |  |  |  |  |
| NPR 2012 | 7.90 | 0.05 | 25252 | 319785 |  | 9.04 | 0.06 | 24262 | 268253 |  | 9.68 | 0.08 | 13277 | 137117 |  | 7.47 | 0.09 | 5952 | 79670 |  |  |  |  |  |

* Chiropractor or physiotherapist visit

**GP visit or chiropractor or physiotherapist visit

Note: The means in in the survey of health and living conditions are weighted. The standard errors (S.E.) are based on a binomial distribution. In the survey of health and living conditions the standard errors are also corrected for clustered sampling.

*A.2: Prevalence and health service use for musculoskeletal disorders by age in women*

|  | <10 |  |  |  |  | 10-19 |  |  |  |  | 20-29 |  |  |  |  | 30-39 |  |  |  |  | 40-49 |  |  |  |
| --- | --- | --- | --- | --- | --- | --- | --- | --- | --- | --- | --- | --- | --- | --- | --- | --- | --- | --- | --- | --- | --- | --- | --- | --- |
|  | *Mean (%)* | *S.E.* | *Freq.* | *N* |  | *Mean (%)* | *S.E.* | *Freq.* | *N* |  | *Mean (%)* | *S.E.* | *Freq.* | *N* |  | *Mean (%)* | *S.E.* | *Freq.* | *N* |  | *Mean (%)* | *S.E.* | *Freq.* | *N* |
| Survey of health and living conditions | | |  |  |  |  |  |  |  |  |  |  |  |  |  |  |  |  |  |  |  |  |  |  |
| 2002 | - | - | - | - |  | 5.03 | 2.09 | 9 | 201 |  | 10.53 | 1.91 | 56 | 531 |  | 14.97 | 1.95 | 98 | 673 |  | 24.57 | 2.47 | 150 | 618 |
| 2005 | - | - | - | - |  | 7.82 | 2.58 | 17 | 220 |  | 10.58 | 1.97 | 52 | 497 |  | 15.32 | 1.99 | 98 | 656 |  | 26.51 | 2.49 | 168 | 640 |
| 2008 | - | - | - | - |  | 7.64 | 2.34 | 18 | 250 |  | 8.24 | 1.86 | 36 | 442 |  | 13.29 | 1.95 | 72 | 582 |  | 23.93 | 2.46 | 133 | 592 |
| 2012 | - | - | - | - |  | 9.60 | 2.08 | 18 | 194 |  | 7.10 | 1.23 | 26 | 402 |  | 15.62 | 1.72 | 57 | 408 |  | 22.04 | 1.89 | 98 | 466 |
| GP visit 2012 | 6.49 | 0.05 | 19372 | 298448 |  | 21.94 | 0.07 | 67840 | 309195 |  | 24.45 | 0.08 | 78251 | 320077 |  | 29.93 | 0.08 | 98709 | 329843 |  | 39.21 | 0.08 | 137971 | 351924 |
| Chiro/Phys 2012* | 2.70 | 0.03 | 8066 | 298448 |  | 6.49 | 0.04 | 20074 | 309195 |  | 10.54 | 0.05 | 33737 | 320077 |  | 16.01 | 0.06 | 52811 | 329843 |  | 19.44 | 0.07 | 68408 | 351924 |
| GP/Chiro/Phys 2012** | 8.72 | 0.05 | 26014 | 298448 |  | 24.74 | 0.08 | 76508 | 309195 |  | 29.42 | 0.08 | 94153 | 320077 |  | 36.90 | 0.08 | 121718 | 329843 |  | 45.83 | 0.08 | 161297 | 351924 |
| NPR 2012 | 0.75 | 0.02 | 2222 | 298448 |  | 2.96 | 0.03 | 9157 | 309195 |  | 3.53 | 0.03 | 11290 | 320077 |  | 4.93 | 0.04 | 16273 | 329843 |  | 7.71 | 0.05 | 27119 | 351924 |
|  | 50-59 |  |  |  |  | 60-69 |  |  |  |  | 70-79 |  |  |  |  | 80+ |  |  |  |  |  |  |  |  |
| Survey of health and living conditions | | |  |  |  |  |  |  |  |  |  |  |  |  |  |  |  |  |  |  |  |  |  |  |
| 2002 | 39.58 | 2.93 | 217 | 565 |  | 45.30 | 3.66 | 169 | 378 |  | 46.12 | 4.22 | 131 | 286 |  | 52.71 | 5.56 | 88 | 165 |  |  |  |  |  |
| 2005 | 37.84 | 2.97 | 203 | 543 |  | 44.78 | 3.47 | 186 | 420 |  | 53.61 | 4.70 | 124 | 231 |  | 55.69 | 5.66 | 88 | 158 |  |  |  |  |  |
| 2008 | 32.27 | 2.81 | 176 | 559 |  | 44.42 | 3.32 | 200 | 457 |  | 45.71 | 4.54 | 113 | 247 |  | 47.59 | 5.58 | 78 | 164 |  |  |  |  |  |
| 2012 | 31.47 | 2.03 | 165 | 524 |  | 43.37 | 2.36 | 188 | 439 |  | 52.49 | 2.91 | 152 | 294 |  | 58.89 | 4.39 | 74 | 126 |  |  |  |  |  |
| GP visit 2012 | 44.85 | 0.09 | 138321 | 308391 |  | 45.35 | 0.10 | 121096 | 267000 |  | 47.39 | 0.13 | 75927 | 160208 |  | 45.68 | 0.13 | 64822 | 141913 |  |  |  |  |  |
| Chiro/Phys 2012* | 20.74 | 0.07 | 63959 | 308391 |  | 20.91 | 0.08 | 55818 | 267000 |  | 20.89 | 0.10 | 33475 | 160208 |  | 12.57 | 0.09 | 17839 | 141913 |  |  |  |  |  |
| GP/Chiro/Phys 2012** | 50.35 | 0.09 | 155286 | 308391 |  | 50.35 | 0.10 | 134425 | 267000 |  | 51.73 | 0.12 | 82881 | 160208 |  | 47.90 | 0.13 | 67979 | 141913 |  |  |  |  |  |
| NPR 2012 | 10.61 | 0.06 | 32722 | 308391 |  | 12.51 | 0.06 | 33412 | 267000 |  | 14.05 | 0.09 | 22504 | 160208 |  | 9.06 | 0.08 | 12852 | 141913 |  |  |  |  |  |

* Chiropractor or physiotherapist visit

**GP visit or chiropractor or physiotherapist visit

Note: The means in in the survey of health and living conditions are weighted. The standard errors (S.E.) are based on a binomial distribution. In the survey of health and living conditions the standard errors are also corrected for clustered sampling.

*A.3: Prevalence and health service use for lower back and neck pain by age in men*

|  | <10 |  |  |  |  | 10-19 |  |  |  |  | 20-29 |  |  |  |  | 30-39 |  |  |  |  | 40-49 |  |  |  |
| --- | --- | --- | --- | --- | --- | --- | --- | --- | --- | --- | --- | --- | --- | --- | --- | --- | --- | --- | --- | --- | --- | --- | --- | --- |
|  | *Mean (%)* | *S.E.* | *Freq.* | *N* |  | *Mean (%)* | *S.E.* | *Freq.* | *N* |  | *Mean (%)* | *S.E.* | *Freq.* | *N* |  | *Mean (%)* | *S.E.* | *Freq.* | *N* |  | *Mean (%)* | *S.E.* | *Freq.* | *N* |
| Survey of health and living conditions | | | |  |  |  |  |  |  |  |  |  |  |  |  |  |  |  |  |  |  |  |  |  |
| 2002 | - | - | - | - |  | 3.67 | 1.78 | 8 | 224 |  | 4.68 | 1.32 | 25 | 529 |  | 7.47 | 1.45 | 49 | 666 |  | 9.64 | 1.62 | 64 | 672 |
| 2005 | - | - | - | - |  | 1.28 | 0.97 | 3 | 253 |  | 2.87 | 1.06 | 15 | 518 |  | 7.29 | 1.45 | 45 | 639 |  | 9.22 | 1.65 | 55 | 613 |
| 2008 | - | - | - | - |  | 3.14 | 1.70 | 6 | 203 |  | 3.62 | 1.23 | 16 | 456 |  | 5.31 | 1.32 | 25 | 528 |  | 9.75 | 1.66 | 59 | 631 |
| 2012 | - | - | - | - |  | 0.00 | 0.00 | 0 | 181 |  | 4.67 | 0.94 | 16 | 417 |  | 7.16 | 1.23 | 30 | 429 |  | 7.58 | 1.17 | 36 | 496 |
| GP visit 2012 | 0.77 | 0.02 | 2422 | 313388 |  | 3.68 | 0.03 | 12054 | 327534 |  | 10.06 | 0.05 | 33481 | 332710 |  | 14.71 | 0.06 | 51102 | 347331 |  | 19.03 | 0.06 | 71013 | 373083 |
| Chiro/Phys 2012* | 2.53 | 0.03 | 7925 | 313388 |  | 2.65 | 0.03 | 8668 | 327534 |  | 5.50 | 0.04 | 18298 | 332710 |  | 8.74 | 0.05 | 30341 | 347331 |  | 10.15 | 0.05 | 37858 | 373083 |
| GP/Chiro/Phys 2012** | 3.21 | 0.03 | 10074 | 313388 |  | 5.81 | 0.04 | 19041 | 327534 |  | 14.11 | 0.06 | 46930 | 332710 |  | 20.97 | 0.07 | 72836 | 347331 |  | 26.17 | 0.07 | 97622 | 373083 |
| NPR 2012 | 0.03 | 0.00 | 104 | 313388 |  | 0.18 | 0.01 | 576 | 327534 |  | 0.57 | 0.01 | 1898 | 332710 |  | 1.15 | 0.02 | 3980 | 347331 |  | 1.69 | 0.02 | 6297 | 373083 |
|  | 50-59 |  |  |  |  | 60-69 |  |  |  |  | 70-79 |  |  |  |  | 80+ |  |  |  |  |  |  |  |  |
| Survey of health and living conditions | | | |  |  |  |  |  |  |  |  |  |  |  |  |  |  |  |  |  |  |  |  |  |
| 2002 | 10.54 | 1.80 | 62 | 593 |  | 13.12 | 2.49 | 50 | 379 |  | 15.93 | 3.38 | 40 | 245 |  | 9.83 | 4.40 | 11 | 102 |  |  |  |  |  |
| 2005 | 11.97 | 1.88 | 70 | 599 |  | 10.70 | 2.12 | 43 | 419 |  | 10.64 | 2.81 | 26 | 246 |  | 6.27 | 3.22 | 7 | 114 |  |  |  |  |  |
| 2008 | 10.63 | 1.88 | 55 | 536 |  | 8.62 | 1.82 | 38 | 466 |  | 7.31 | 2.54 | 17 | 223 |  | 8.16 | 3.52 | 11 | 129 |  |  |  |  |  |
| 2012 | 9.39 | 1.23 | 44 | 514 |  | 10.09 | 1.37 | 44 | 459 |  | 10.59 | 1.99 | 24 | 233 |  | 12.08 | 3.62 | 9 | 78 |  |  |  |  |  |
| GP visit 2012 | 20.82 | 0.07 | 66591 | 319785 |  | 18.32 | 0.08 | 49145 | 268253 |  | 15.42 | 0.10 | 21136 | 137117 |  | 14.96 | 0.13 | 11917 | 79670 |  |  |  |  |  |
| Chiro/Phys 2012* | 9.54 | 0.05 | 30496 | 319785 |  | 8.36 | 0.05 | 22416 | 268253 |  | 7.11 | 0.07 | 9752 | 137117 |  | 4.17 | 0.07 | 3320 | 79670 |  |  |  |  |  |
| GP/Chiro/Phys 2012** | 27.33 | 0.08 | 87399 | 319785 |  | 24.07 | 0.08 | 64576 | 268253 |  | 20.34 | 0.11 | 27887 | 137117 |  | 17.55 | 0.13 | 13980 | 79670 |  |  |  |  |  |
| NPR 2012 | 1.91 | 0.02 | 6111 | 319785 |  | 1.74 | 0.03 | 4654 | 268253 |  | 1.64 | 0.03 | 2253 | 137117 |  | 1.26 | 0.04 | 1001 | 79670 |  |  |  |  |  |

* Chiropractor or physiotherapist visit

**GP visit or chiropractor or physiotherapist visit

Note: The means in in the survey of health and living conditions are weighted. The standard errors (S.E.) are based on a binomial distribution. In the survey of health and living conditions the standard errors are also corrected for clustered sampling.

*A.4: Prevalence and health service use for lower back and neck pain by age in women*

|  | <10 |  |  |  |  | 10-19 |  |  |  |  | 20-29 |  |  |  |  | 30-39 |  |  |  |  | 40-49 |  |  |  |
| --- | --- | --- | --- | --- | --- | --- | --- | --- | --- | --- | --- | --- | --- | --- | --- | --- | --- | --- | --- | --- | --- | --- | --- | --- |
|  | *Mean (%)* | *S.E.* | *Freq.* | *N* |  | *Mean (%)* | *S.E.* | *Freq.* | *N* |  | *Mean (%)* | *S.E.* | *Freq.* | *N* |  | *Mean (%)* | *S.E.* | *Freq.* | *N* |  | *Mean (%)* | *S.E.* | *Freq.* | *N* |
| Survey of health and living conditions | | | |  |  |  |  |  |  |  |  |  |  |  |  |  |  |  |  |  |  |  |  |  |
| 2002 | - | - | - | - |  | 1.41 | 1.00 | 2 | 201 |  | 2.39 | 0.92 | 12 | 531 |  | 5.36 | 1.23 | 35 | 673 |  | 6.10 | 1.37 | 37 | 618 |
| 2005 | - | - | - | - |  | 3.42 | 1.69 | 7 | 220 |  | 5.32 | 1.43 | 26 | 497 |  | 5.55 | 1.27 | 36 | 656 |  | 9.24 | 1.64 | 59 | 640 |
| 2008 | - | - | - | - |  | 2.77 | 1.49 | 7 | 250 |  | 3.60 | 1.27 | 16 | 442 |  | 5.28 | 1.27 | 28 | 582 |  | 10.43 | 1.76 | 59 | 592 |
| 2012 | - | - | - | - |  | 2.86 | 1.14 | 5 | 194 |  | 2.54 | 0.78 | 10 | 402 |  | 7.28 | 1.21 | 26 | 408 |  | 8.74 | 1.30 | 40 | 466 |
| GP visit 2012 | 0.76 | 0.02 | 2271 | 298448 |  | 5.23 | 0.04 | 16161 | 309195 |  | 12.92 | 0.06 | 41365 | 320077 |  | 18.92 | 0.07 | 62415 | 329843 |  | 24.80 | 0.07 | 87281 | 351924 |
| Chiro/Phys 2012* | 1.97 | 0.03 | 5874 | 298448 |  | 3.54 | 0.03 | 10947 | 309195 |  | 7.95 | 0.05 | 25431 | 320077 |  | 12.25 | 0.06 | 40417 | 329843 |  | 13.08 | 0.06 | 46036 | 351924 |
| GP/Chiro/Phys 2012** | 2.65 | 0.03 | 7905 | 298448 |  | 7.86 | 0.05 | 24313 | 309195 |  | 18.56 | 0.07 | 59404 | 320077 |  | 27.31 | 0.08 | 90090 | 329843 |  | 33.35 | 0.08 | 117375 | 351924 |
| NPR 2012 | 0.03 | 0.00 | 82 | 298448 |  | 0.25 | 0.01 | 777 | 309195 |  | 0.76 | 0.02 | 2422 | 320077 |  | 1.37 | 0.02 | 4529 | 329843 |  | 1.91 | 0.02 | 6724 | 351924 |
|  | 50-59 |  |  |  |  | 60-69 |  |  |  |  | 70-79 |  |  |  |  | 80+ |  |  |  |  |  |  |  |  |
| Survey of health and living conditions | | | |  |  |  |  |  |  |  |  |  |  |  |  |  |  |  |  |  |  |  |  |  |
| 2002 | 8.15 | 1.63 | 45 | 565 |  | 10.05 | 2.19 | 37 | 378 |  | 9.06 | 2.39 | 25 | 286 |  | 14.42 | 3.79 | 22 | 165 |  |  |  |  |  |
| 2005 | 7.13 | 1.59 | 39 | 543 |  | 7.15 | 1.80 | 30 | 420 |  | 6.28 | 2.25 | 14 | 231 |  | 10.41 | 3.44 | 16 | 158 |  |  |  |  |  |
| 2008 | 9.94 | 1.77 | 53 | 559 |  | 7.56 | 1.73 | 33 | 457 |  | 4.95 | 1.96 | 12 | 247 |  | 9.72 | 3.32 | 16 | 164 |  |  |  |  |  |
| 2012 | 10.28 | 1.34 | 55 | 524 |  | 9.51 | 1.37 | 40 | 439 |  | 10.47 | 1.77 | 30 | 294 |  | 12.70 | 2.80 | 14 | 126 |  |  |  |  |  |
| GP visit 2012 | 25.96 | 0.08 | 80069 | 308391 |  | 20.79 | 0.08 | 55515 | 267000 |  | 19.68 | 0.10 | 31522 | 160208 |  | 18.53 | 0.10 | 26291 | 141913 |  |  |  |  |  |
| Chiro/Phys 2012* | 11.75 | 0.06 | 36251 | 308391 |  | 9.60 | 0.06 | 25623 | 267000 |  | 7.54 | 0.07 | 12077 | 160208 |  | 3.48 | 0.05 | 4934 | 141913 |  |  |  |  |  |
| GP/Chiro/Phys 2012** | 33.37 | 0.08 | 102920 | 308391 |  | 26.96 | 0.09 | 71982 | 267000 |  | 24.35 | 0.11 | 39013 | 160208 |  | 20.37 | 0.11 | 28907 | 141913 |  |  |  |  |  |
| NPR 2012 | 1.88 | 0.02 | 5800 | 308391 |  | 1.71 | 0.03 | 4575 | 267000 |  | 1.93 | 0.03 | 3089 | 160208 |  | 1.30 | 0.03 | 1845 | 141913 |  |  |  |  |  |

* Chiropractor or physiotherapist visit

**GP visit or chiropractor or physiotherapist visit

Note: The means in in the survey of health and living conditions are weighted. The standard errors (S.E.) are based on a binomial distribution. In the survey of health and living conditions the standard errors are also corrected for clustered sampling.
